# Antiseizure Medication-Induced Nystagmus During Eye Closure Identified by Electroencephalography

**DOI:** 10.7759/cureus.56884

**Published:** 2024-03-25

**Authors:** Ryota Sasaki, Mirei Hara, Nagisa Yasuda, Nahomi Osugi, Ichiro Nakagawa

**Affiliations:** 1 Department of Neurosurgery, Nara Medical University, Kashihara, JPN; 2 Department of Clinical Laboratory, National Hospital Organization Nara Medical Center, Nara, JPN

**Keywords:** nystagmus, epilepsy, electrooculogram, electroencephalogram, adverse effect

## Abstract

Nystagmus is a well-known side effect of antiseizure medicines (ASMs), but it is often underestimated and overlooked. Here, we describe a case in which nystagmus during eye closure was identified early using routine electroencephalography (EEG). A 34-year-old man developed focal epilepsy after head trauma at the age of 25 years. The patient was treated with carbamazepine but liver dysfunction was observed; therefore, treatment was attempted with lacosamide (LCM) and lamotrigine. With an increase in the LCM dose, steep potential changes suggestive of horizontal nystagmus were observed in the electrooculogram, F7, and F8 on EEG, and the patient complained of eye shaking during eye closure. These symptoms and EEG findings improved with LCM dose reduction. If the presence of nystagmus is identified on EEG coincidentally and a patient’s subjective symptoms with ASM are confirmed, it is advisable to taper and/or discontinue the causative agent.

## Introduction

For the treatment of epilepsy, appropriate antiseizure medicine (ASM) should be administered based on the type of epilepsy, etiology, and comorbidities. However, clinicians should be aware that the administration of ASM may lead to an adverse effect (AE), which can significantly affect a patient’s quality of life [1]. Among the various AEs, nystagmus is one of the most frequently encountered but easily missed symptoms. Here, we report a case of nystagmus with closed eyes that developed with increasing doses of lacosamide (LCM) and was identified by routine outpatient electroencephalography (EEG).

## Case presentation

**Figure 1:**
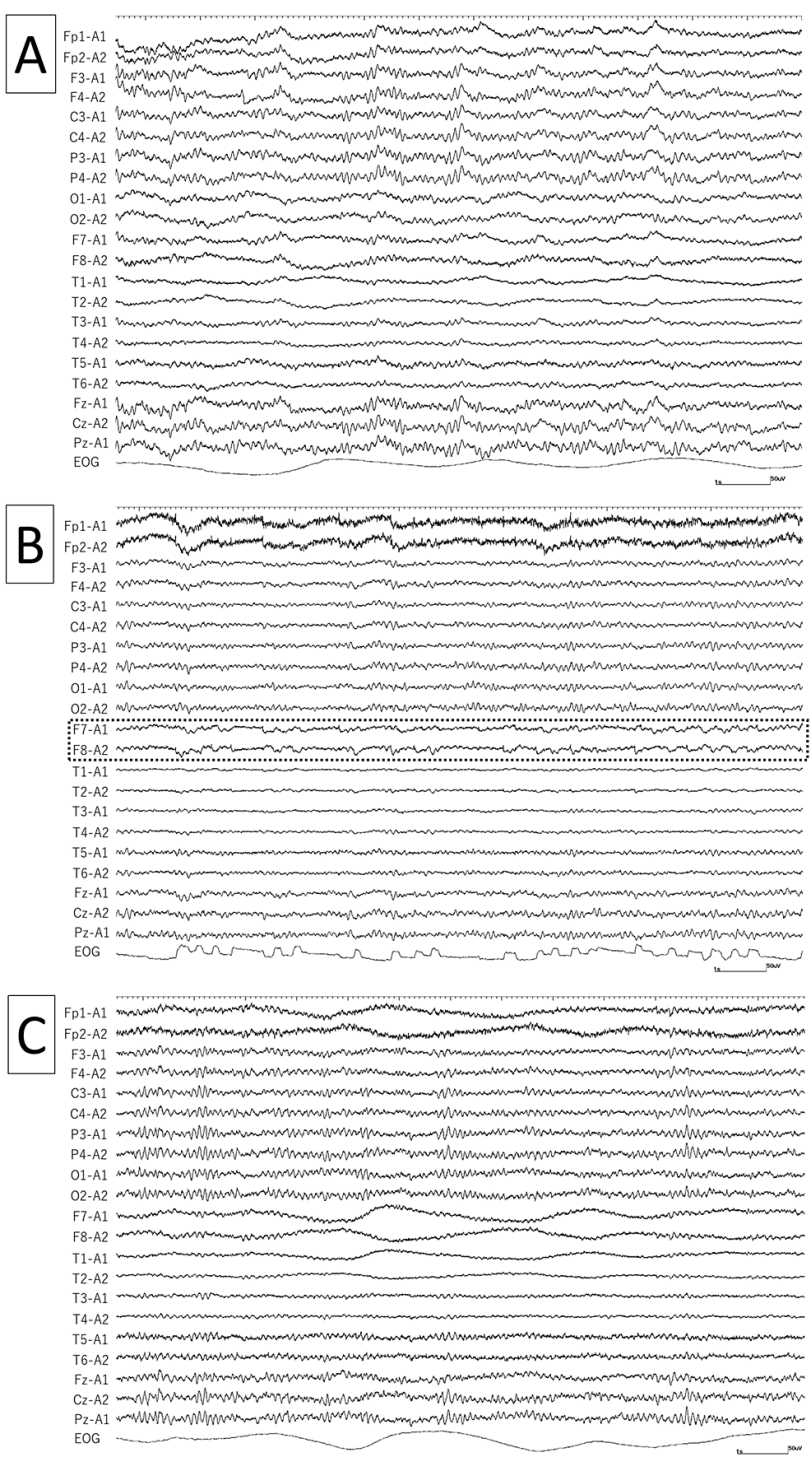
Changes in interictal electroencephalogram (EEG) findings over time. At our institution, electrooculogram (EOG) electrodes are placed in the left inferior lateral orbit (PG1) and right superior lateral orbit (PG2), and EOG is measured at the PG1-PG2 bipolar montage. For example, if the eye turns to the right, EOG records a negative potential. (A) At the initial visit, there was no obvious nystagmus when his eyes were closed. (B) EEG during lacosamide (LCM) 200 mg and lamotrigine (LTG) 100 mg. When his eyes were closed, EOG showed a steep potential change that was suspicious of nystagmus. In F7 and F8, symmetric potential changes with some lateral rectus spikes were persistently observed, which corresponded to the EOG potential changes (dotted line). (C) EEG during LCM 100 mg and LTG 150 mg. Potential changes associated with nystagmus during eye closure disappeared. Sampling rate: 500 Hz, sensitivity 10 mv, high-frequency filter: 120 Hz, and time constant: 0.3 s.

A 34-year-old right-handed man suffered a right temporal lobe contusion after a fall at the age of 25 years and developed focal epilepsy six months later. The patient had no obvious neurological abnormalities or nystagmus. Simple auditory hallucinations were observed in focal awareness seizures, which subsequently led to focal impaired awareness seizures (FIAS). EEG showed slow waves in the right temporal area, but no nystagmus (Figure 1, Panel A). He had been seizure-free on carbamazepine (CBZ) monotherapy for four years, but the drug-induced liver dysfunction worsened over time, forcing a change to another ASM. After discontinuing CBZ, he was administered lamotrigine (LTG) alone, but during the dose increase to 100 mg/day, FIAS recurred; therefore, LCM was added. The dose was increased to 100 mg/day of LTG and 200 mg/day of LCM, and seizures were controlled (blood concentration LCM 5.56 µg/mL, LTG 2.35 µg/mL). However, in the follow-up EEG three months after changing ASM, steep changes in potential were observed during eye closure in the electrooculogram (EOG), which had not been previously observed and were suspected to be nystagmus (Figure 1, Panel B).

In addition, symmetric potential changes with some lateral rectus spikes were continuously observed at F7 and F8, which also corresponded to the EOG, and horizontal nystagmus was suspected. Subsequently, when confirming the patient’s symptoms, he complained that he had tolerated them, but that his eyes shook and felt uncomfortable, especially when they were closed. He did not complain of other symptoms such as drowsiness and unsteadiness. When he was switched to LTG 150 mg/day and LCM 100 mg/day (blood concentration LCM 3.81 µg/mL, LTG 5.81 µg/mL), his subjective nystagmus disappeared, and the EEG one year after changing ASM did not show any potential changes indicative of nystagmus (Figure 1, Panel C). Thereafter, the liver function abnormalities also improved, and no seizures have been observed for the past four years.

## Discussion

Nystagmus due to ASM is induced by Na channel blockers such as phenytoin, CBZ, LTG, oxcarbazepine, and LCM [2,3]. The mechanism by which nystagmus occurs is unknown, but animal studies suggest that it is caused by the impairment of voltage-gated Na channel blockers in the Purkinje cells of the cerebellum and that both horizontal and vertical nystagmus could occur [4]. Although the incidence increases in a dose-dependent manner, it is a reversible change that improves with titration and/or discontinuation. Therefore, it is important to diagnose early and adjust the volume of ASM [3].

Nystagmus is a familiar symptom to those who perform neurological examinations, but visual identification is sometimes difficult or only appears with the eyes closed without a spontaneous complaint from the patient. Therefore, tools such as Frenzel glasses or electronystagmography are sometimes necessary [5,6]. However, they are difficult to use without an otolaryngologist. The findings of this case indicate that EEG can be used to determine the efficacy of treatment for epilepsy as well as to identify the presence of nystagmus. The surface of the cornea has a positive potential; when the cornea turns toward a nearby electrode during nystagmus, the potential is recorded, and eye movements can be captured even when the eyes are closed [7]. Although EOG was recorded in all cases at our institution, similar potential changes can be recorded at F7 and F8, and evaluation of nystagmus is possible using only the 10-20 system. Although it is necessary to distinguish nystagmus from slow waves of cerebral cortex origin, it can be judged from the symmetrical potential changes of F7 and F8, the steep potential changes reflecting the rapid phase of the saccade, and the presence of lateral rectus spikes. In this case, vertical nystagmus was not observed, but if it were, synchronized potential changes would have occurred at Fp1 and Fp2. Our findings may be applied to the early identification of nystagmus not only in patients who can communicate but also in mentally challenged patients who have difficulty reporting symptoms.

## Conclusions

As increasing the dosage of ASM may cause horizontal nystagmus with eyes closed, care should be taken. Nystagmus can often be seen in a routine EEG, and the EEG reader should make a notation in the interpretation that nystagmus is seen, suggestive of high levels of ASM. If ASM-induced nystagmus can be identified on EEG, the ASM dosage should be reconsidered after reassessing the subjective symptoms.
